# Supplementary material for: Association between heavy metal exposure and bacterial vaginosis: A cross-sectional study
Source: PLoS One. 2025 Jan 8;20(1):e0316927. doi: 10.1371/journal.pone.0316927 (PMC11709292; doi:10.1371/journal.pone.0316927)
Supplement: S1 Table — (DOCX) [file pone.0316927.s001.docx]

**Supplementary Table 1 Sensitivity analysis of heavy metals concerning BV**

|  | **Unadjusted Model** | **Model I** | **Model II** |
| --- | --- | --- | --- |
| **Outcome** | **OR (95%Cl) P value** | **OR (95%Cl) P value** | **OR (95%Cl) P value** |
| **Serum lead (ug/dl)** |  |  |  |
| **Overall** | 1.22 (1.13, 1.31) <0.001 | 1.15 (1.06, 1.24) <0.001 | 1.11 (1.03, 1.20) 0.005 |
| **Q1 (0.2 to 0.7)** | 1.0 [Reference] | 1.0 [Reference] | 1.0 [Reference] |
| **Q2 (0.8 to 1.2)** | 1.29 (1.05, 1.58) 0.014 | 1.19 (0.96, 1.48) 0.107 | 1.13 (0.91, 1.41) 0.270 |
| **Q3 (1.3 to 23.9)** | 1.77 (1.45, 2.16) <0.001 | 1.52 (1.21, 1.91) <0.001 | 1.39 (1.10, 1.76) 0.006 |
| **Serum total mercury (ug/l)** |  |  |  |
| **Overall** | 0.99 (0.95, 1.04) 0.672 | 0.99 (0.95, 1.04) 0.682 | 1.00 (0.95, 1.05) 0.898 |
| **Q1 (0.07 to 0.4)** | 1.0 [Reference] | 1.0 [Reference] | 1.0 [Reference] |
| **Q2 (0.5 to 1.1)** | 1.10 (0.90, 1.35) 0.342 | 1.02 (0.83, 1.26) 0.863 | 1.02 (0.82, 1.27) 0.857 |
| **Q3 (1.2 to 27.4)** | 1.00 (0.82, 1.24) 0.968 | 0.91 (0.73, 1.14) 0.428 | 0.94 (0.74, 1.18) 0.581 |
| **Serum cadmium (ug/l)** |  |  |  |
| **Overall** | 1.53 (1.32, 1.77) <0.001 | 1.57 (1.35, 1.84) <0.001 | 1.45 (1.24, 1.70) <0.001 |
| **Q1 (＜0.2)** | 1.0 [Reference] | 1.0 [Reference] | 1.0 [Reference] |
| **Q2 (0.2 to 0.4)** | 1.03 (0.77, 1.37) 0.856 | 0.95 (0.70, 1.28) 0.720 | 1.00 (0.74, 1.36) 0.985 |
| **Q3 (0.5 to 7.4)** | 1.59 (1.18, 2.13) 0.002 | 1.48 (1.08, 2.03) 0.014 | 1.43 (1.03, 1.96) 0.031 |

Unadjusted model: no covariates were adjusted.

Model I: Part of covariates, age, race, education, and BMI were adjusted.

Mode II: All covariates, age, race, education, BMI, moderate activity over the past 30 days, PIR, marital status, HDL-cholesterol, total cholesterol, total number of people in the household, total calcium, uric acid, and vigorous activity over the past 30 days were adjusted.

Abbreviations: BMI, Body Mass Index; PIR, Poverty Income Ratio; HDL, High-Density Lipoprotein; CI, confidence interval; OR, odds ratio.
