# Supplementary material for: Association between heavy metal exposure and bacterial vaginosis: A cross-sectional study
Source: PLoS One. 2025 Jan 8;20(1):e0316927. doi: 10.1371/journal.pone.0316927 (PMC11709292; doi:10.1371/journal.pone.0316927)
Supplement: Attachment — Submitted filename: Response to reviewers.pdf [file pone.0316927.s004.pdf]

Responses to reviewers' comments

Dear reviewers.

We appreciate your professional review of our article It is with excitement that I resubmit to you a revised version of the manuscript (Manuscript No.: PONE-D-24-18888R1). Thank you for giving me the opportunity to revise and resubmit this manuscript. As you know, several problems need to be addressed. Your comments have greatly improved the quality of our article.

We have carefully reviewed the comments and have revised the manuscript accordingly. I have responded specifically to each suggestion below, beginning with your own. Our responses are given in a point-by-point manner below. To make the changes easier to identify where necessary, I have numbered them.

The specific comments from the reviewers are highlighted in red font, while my responses are in black.

To Reviewer 2

1.When referring to "serum heavy metal exposure" it is understood as if the serum would be the one exposed. This expression is incorrect because it is the individual who has been exposed to such metals and this is reflected by elevated serum levels. But it is not the serum that is exposed (line 45).

Thanks for your suggestions. We feel sorry for the improper wording. Based on your comments, we have made the corrections to harmonize the unit within the abstract. We have replaced serum heavy metal exposure with heavy metal exposure to make the presentation of the statements in the article more scientific and to avoid ambiguity (line 45).

2. Improve the epidemiological data in citation 3 with more information provided by an agency such as the World Health Organization or another agency that provides more recent information, such as geographic location, age, or any important clinical condition.

I very much appreciate the time and effort you've put into your comments. We agree with the reviewer's comment concerning this issue. We improve the epidemiological data in citation 3 with more information provided by an agency such as the World Health Organization or another agency that provides more recent information, such as geographic location, age, or any important clinical condition (lines 72 to 76).

3. Indicate the consequences of disturbing the "equilibrium of human reproductive hormones" (line 89).

We acknowledge your comments very much, which are valuable in improving the quality of our manuscript. We indicated the consequences of disturbing the "equilibrium of human reproductive hormones" to make the article more readable and scientific (lines 91 to 92).

4. Search for more current references instead of 35 and 36.

Thanks for the suggestion, your suggestion means a lot to us. We searched for more current references instead of 35 and 36 (line 99).

5.Lines 320 to 324 do not account for the time constraint of the data collected. Indicate what is stated in your response in the text.
I appreciate the time and effort you've put into your comments. As you suggested, I have indicated what is stated in your response in the text (lines 345 to 348).

6. You indicate that the covariates were initially established with a literature review and international practice standards, but you do not cite them in the text. They should be placed in the introduction to emphasize the importance of the study.
We thank the reviewers for their constructive criticism, and time spent analyzing this manuscript. I have placed the literature review and international practice standards in the introduction to emphasize the importance of the study (lines 103 to 108).

7. Indicate in the text the reasons why the data needed to be adjusted with the appropriate references and as explained in the response to the review.
We thank the reviewer for the kind comments. We have indicated in the text the reasons why the data needed to be adjusted with the appropriate references (lines 168 to 173). Confounders refer to all the factors (both known and unknown) other than the study factors that may affect the outcome. Ensuring the authenticity and reliability of the study results is the greatest significance of controlling for confounders. Confounding was controlled by the inclusion of covariates, thereby reducing false-positive results to increase the fidelity of the study.

8. Based on the principle that OR is a measure of association between exposure and event (OR=odds that a case was exposed/odds that a control was exposed), data from persons exposed and not exposed to heavy metals and persons who did and did not develop BV are always required to calculate the OR. With the information they have they should calculate only the possible odds. Review the basis for epidemiological calculations. Other more relevant epidemiological data such as prevalences could also be calculated.
We are very grateful for your comments on the manuscript. Thanks to your suggestion and correction of our error, we reviewed the epidemiological basis, confirmed the definition of OR (correlation between exposure factors and the occurrence of event outcomes), and applied logistic regression to calculate ORs to obtain the degree of association between illness and exposure. The feasibility of this OR calculation has been demonstrated in a number of previous cross-sectional studies using data from the NHANES database. The prevalence of BV has been previously calculated using data from the NHANES database from 2001 to 2004. We have cited this study in our article (lines 74 to 76).

9. Include the percentage of the eligible population in the text.
We thank you very much for your comments for pointing out this omission. As per your

suggestion, we included the percentage of the eligible population in the text (lines 138).

10. Indicate in the text why group analyses are performed when individual data are available. How they have responded in the review.
We are very grateful for your comments and thoughtful suggestions. We have already indicated in the text why group analyses are performed when individual data are available and provided an overview of the NHANES database in our article: the survey examines a nationally representative sample of about 5,000 people each year, selecting the survey sample to represent the U.S. population of all ages. Individuals are sampled to represent the group population (lines 122 to 127).

11. Analyze in depth whether the statistical and epidemiological estimates provided are adequate according to the data they have and consider possible important changes.
Thanks for your valuable suggestions. We understand your concern about this issue. In terms of statistical methods, we used multivariate logistic regression analyses to explore the relationship between exposure factors and disease, stratified analyses to explore what was happening in different subgroups, multiple interpolation methods, and sensitivity analyses to further validate the scientific validity of the findings, and finally curve fitting to visualize the relationship between exposure and outcomes. In addition, we reviewed a large amount of literature and discussed the endocrine and immune systems to explain and support our findings more convincingly. We have described the possible important changes and limitations in the text.

I appreciate the time and detail provided by each reviewer and have incorporated the suggested changes into the manuscript to the best of my ability. The manuscript has certainly benefited from these insightful revision suggestions. I hope that the changes I've made resolve all your concerns about the article. I'm more than happy to make any further changes that will improve the paper and facilitate successful publication.

Sincerely thank you!

Yu-Xue Feng
fyxgzhmu1226@outlook.com
