# Supplementary material for: Association between heavy metal exposure and bacterial vaginosis: A cross-sectional study
Source: PLoS One. 2025 Jan 8;20(1):e0316927. doi: 10.1371/journal.pone.0316927 (PMC11709292; doi:10.1371/journal.pone.0316927)
Supplement: Attachment — Submitted filename: Response Letter.docx [file pone.0316927.s002.docx]

Dear Editor,

Thank you for your positive comment on our manuscript. I have submitted the Human Participants Research Checklist in the submission system. I am excited to resubmit a revised manuscript version: Association between heavy metal exposure and bacterial vaginosis: a cross-sectional study for the Scientific Reports. Readers have been learning a lot from all the papers published in your journal. Hopefully, we can have our article considered for publication in your journal. Please feel free to contact us for any other corrections we could make. Thank you again for allowing me to revise and resubmit this manuscript.

Sincerely,

Yu-Xue Feng

fyxgzhmu1226@outlook.com
