# Supplementary material for: Association between heavy metal exposure and bacterial vaginosis: A cross-sectional study
Source: PLoS One. 2025 Jan 8;20(1):e0316927. doi: 10.1371/journal.pone.0316927 (PMC11709292; doi:10.1371/journal.pone.0316927)
Supplement: Attachment — Submitted filename: Responses to reviews.docx [file pone.0316927.s003.docx]

Responses to reviewers' comments

Dear reviewers.

Firstly, we would like to thank you for your kind letter and for the reviewers’ constructive comments concerning our article (Manuscript No.: PONE-D-24-18888). These comments are all valuable and helpful for improving our article. All the authors have seriously discussed all these comments. According to the reviewers’ comments, we have tried to modify our manuscript to meet your journal's requirements.

In response to your suggestions, we have implemented a series of significant alterations and enhancements to the article to optimize its quality and clarity. The following is a summary of the principal modifications we have incorporated based on your feedback and implemented in the article. In addition, we have changed the author's affiliation. Point-by-point responses to the reviewers are listed below this letter.

The specific comments from the reviewers are highlighted in red font, while my responses are in black.

**To Reviewer 1**

1. Data from almost two decades back on the prevalence of heavy metals may be extrapolated to the current health condition.

Thank you for your advice, data on heavy metals for this study were collected from the NHANES database from 2001 to 2004, which reflected the health condition and cannot be extrapolated to the current prevalence. We have clarified the limitation in the discussion on lines 323 to 336.

2. It may be clarified whether any earlier research on the correlation or causation of heavy metals has been done on the same dataset or not, and whether this is an entirely fresh examination. Since the data were collected, the supposition would be that they were also examined. It may also be clarified if the earlier studies were different in their approach and results, and whether this paper does indeed present some new insights.

Thanks for the suggestion, your suggestion means a lot to us. The data on BV were obtained from the NHANES, there was no earlier research on the correlation or causation of heavy metals has been done on the same dataset and this is an entirely fresh examination. In the study, multivariable logistic regression models were utilized to assess the correlation between serum heavy metal exposure and BV, a stratified analysis was performed to investigate the relationship among different population groups further, and smooth curve fittings were conducted to intuitively evaluate the correlation. The cross-sectional study unveiled a substantial correlation between serum Pb and Cd with elevated susceptibility to BV. It is the first study that demonstrated the correlation between heavy metals and the risk of BV, and this point has been emphasized in the discussion (lines 259 to 261).

3. An inherent limitation is the pathway between the heavy metals and BV. It is not clear from the paper which of the pathways are the most likely in this study and which need to be addressed by regulatory agencies as recommended in the paper.

Thank you for your valuable advice, it is a cross-sectional study that could explore the correlation between heavy metals and the risk of BV and could not reveal the pathway between the heavy metals and BV. However, women’s endocrine and immune systems may be damaged because of the presence of high concentrations of serum heavy metals and we have explained the potential pathways in the discussion. Meanwhile, we have highlighted the inherent limitation in the discussion on lines 316 to 318.

**To Reviewer 2**

1.The same sentences are repeated in the abstract (lines 34 to 37 and 39 to 41).

We appreciate your correlation. We are sorry for our careless mistakes. We have deleted one of the sentences to make the article more compact and improve its readability.

2. The abstract does not explain the importance of carrying out the study, especially of the serum heavy metal variable, nor the importance of finding the association between the two variables.

Thank you for pointing out this problem in the abstract. The importance of carrying out the study, the serum heavy metal variable, and the importance of finding the association between the two variables have been explained in the introduction in detail. To improve the readability of the article, we explained the above importance briefly in the abstract (lines 43 to 45).

3. The introduction should use current literature and provide epidemiological data to support the statements made.

Thank you for your suggestions regarding our research. We have reviewed the available literature and cited relevant epidemiological studies in the introduction to support the statements made (lines 73 to 75).

4. Reference 25 (line 75) analyzes a population of non-smokers and the results cannot be generalized to the entire population. Cite other articles or reword the sentence and change the terminology "bacterial flora" to microbiota. Explain the meaning of "human equilibrium" (line 78).

Thank you so much for your careful check. We feel sorry for the improper wording. We modified the expression according to reference 26 to emphasize that the restricted population of the study was the non-smokers (lines 85 to 88). We have used “microbiota” as you suggested (lines 87). With references 27 and 28, we have also changed “human equilibrium" to “the equilibrium of human reproductive hormones” (lines 88 to 89).

5. Support lines 82 to 85 with bibliographic references.

Thank you for your review and valuable suggestions. We have support lines 82 to 85 with bibliographic references 32 to 36 (lines 93 to 96).

6. Why were these years chosen to conduct the study and not use data from more recent years? There should be an important justification for considering the analysis of data from more than 20 years ago.

Thank you for pointing out this problem in the manuscript. Due to the limitations of the NHANES database, the available data on bacterial vaginosis (BV) is only accessible for 2001-2004. Therefore, when conducting our analysis, we had to solely rely on the data collected during this specific time frame. We proceeded with the available data to perform an accurate evaluation and draw conclusions regarding BV within this limited period. This limitation has been pointed out in the limitation of the manuscript (lines 323 to 326).

7. Based on what criteria are the covariates established?

Thank you for your review. First, we reviewed the literature to include data on potential covariates. Then, according to international practice standards, we included potential covariates in the basic or full models to observe the changes in regression coefficients. Covariates were included as potential confounders in the final models if they changed the estimates of heavy metals on BV by more than 10% or were significantly associated with BV. We added the covariates in the method and listed references to support them[1, 2].

8. Revise the total cholesterol value for the whole population (does not agree with the average obtained in the subpopulations with positive and negative BV results).

We appreciate your feedback. It is a giant mistake that affects the whole quality of our article. We feel sorry for our carelessness. We have corrected it, and we also appreciate your point. We revised the cholesterol value in the average obtained in the subpopulations with positive and negative BV results.

9. How do you justify using covariates to adjust the data? Is this data adjustment really necessary?

Thank you for your valuable feedback. We reviewed the literature to include data on potential covariates. Moreover, according to international practice standards, we included potential covariates in the basic or full models to observe the changes in regression coefficients. We selected these confounders based on their associations with the outcomes of interest or a change in effect estimate of more than 10%. Covariates were included as potential confounders in the final models if they changed the estimates of heavy metals on BV by more than 10% or were significantly associated with BV. Based on these two criteria, we screened for covariates that could affect the results to make our study as scientific as possible[1, 2].

10. The figures are not clear. There are no legends explaining the meaning of the blue and red dots.

Thank you for your comment regarding the figure. We understand your concerns about the quality of the figures and we have re-uploaded them with greater clarity. In addition, we have added legends to the figures to explain the meaning of the blue and red dots in lines 696 to 701.

11. It is important to consider that the OR calculations require information on people who did and did not have exposure (heavy metals) and people who did and did not develop the event (BV). Only in this way can an association be established.

Thank you for your valuable suggestion, which is indeed pertinent. In fact, our original design idea coincided with yours. However, considering that the NHANES data did not include the population with or without heavy metal exposure, there were only data measuring serum heavy metal concentrations in the population. Therefore, we only had the serum heavy metal concentration as a continuous variable to work with and designed a compartmentalized study methodology for this.

12. No table indicates the population with or without exposure to heavy metals and the prevalence of BV (the main association to be analyzed in this study). The purpose of analytical cross-sectional studies is to determine prevalences and none are detailed here.

Thanks again for your pertinent suggestions for changes. To take up answer 11, due to data limitations, we unfortunately were unable to obtain data on the population with or without heavy metal exposure.

We refer to other well-designed and rigorous literature[3-5] to explore whether the risk of acquiring BV in women increases with higher heavy metal concentrations by dividing the heavy metal serum concentrations in thirds into three intervals, Q1-Q3, and calculating the ORs for Q2 and Q3 using the lowest concentration in the study population as the control group.

13. The conclusion shows that causality has been found and there are results that are not even statistically significant. In addition, the possible confounders that could exist in this analysis are not analyzed.

Thank you for your offer and we apologize for our lack of rigorous language. In lines 332-336, we have corrected ambiguous expressions. The study is a cross-sectional study, and its results can only indicate whether heavy metals are associated with bacterial vaginitis and do not conclude whether there is a causal relationship between exposure and outcome. At the same time, we refer to several papers and include as many recognized confounders as possible as covariates. However limited to the restricted type of data in the NHANES database, we were unable to exclude all confounders. This limitation we have acknowledged inside lines 320 to 323.

14. It is necessary to determine the percentage of the population that was eligible and from which the information was obtained to analyze the data. This helps to determine that there is no over-estimation or under-estimation of the data analyzed and the results obtained. Ideally, the population analyzed should correspond to at least 80% of the eligible population.

We appreciate your valuable feedback. Our research examined a total of 21,161 individuals from the NHANES database who had the potential to be suitable participants. Furthermore, we excluded individuals with incomplete information regarding BV (n=18,355) and serum heavy metal levels (n=99). Following the exclusion of populations lacking crucial covariate data, a total of 2,493 18-year-old to 49-year-old female participants were ultimately enrolled in our research. The population analyzed corresponds to 92% of the eligible population. Additionally, we used multiple interpolations to fill in the missing values of the covariates and used them as sensitivity analyses.

15. The graphs show group data analysis, but in this study, you collected information from each individual, so individual data analysis is essential since this is not an ecological study. Even the use of linear correlation analysis, which should be used only in the analysis of group data, is questionable. The use of correlation in these studies would introduce an ecological bias, because the relationship obtained at the group level may differ from what is determined at the individual level.

Thanks for your careful checks. As the sample design of NHANES described each single year and any combination of consecutive years comprised a nationally representative sample of the population. However, because NHANES went to only a small number of PSUs each year, estimates for single-year data were relatively unstable (large variance estimates). In addition, releasing only 1 year of data increased the possibility of disclosure of a sample person’s identity. As a result, data were publicly released in 2-year cycles. In general, any 2-year data cycle in NHANES can be combined with adjacent 2-year data cycles to create analytic data files based on 4 or more years of data, to produce estimates with greater precision and smaller sampling error.

16. It is necessary to carry out an in-depth analysis of the biases that could interfere with the hypothesis established in this study. Mainly, in the confounding bias, because the existence of heavy metals in the serum could be only a confounder and this would invalidate the whole study. It is necessary to justify this section with criteria used in epidemiology.

Thank you again for your positive comments and valuable suggestions to improve the quality of our manuscript. As answer 9 said, we selected these confounders based on their associations with the outcomes of interest or a change in effect estimate of more than 10%. Covariates were included as potential confounders in the final models if they changed the estimates of heavy metals on BV by more than 10% or were significantly associated with BV. Based on these two criteria, we screened for covariates that could affect the results to make our study as scientific as possible[1, 2]. Moreover, stratified analyses were generated using covariates to alleviate potential bias in the study[6]. In epidemiology, multivariable logistic regression models, a method to eliminate the effects of confounding factors, were utilized in the study to assess the correlation between heavy metals and BV, and all covariates were adjusted in model Ⅱ. We used more covariates to conduct the stratified analyses to recognize that heavy metals are not the confounders and the result of stratified analyses was stable. We modified Table 3 and described the results on lines 238 to 246. Sensitivity analysis was performed after the missing data of covariates were populated by multiple interpolations when the missing data of heavy metals and BV were eliminated.

17. Finally, it is necessary to carefully revise the entire text of the article, improve the wording, use the current bibliography, and revise the wording in English.

Thanks for your suggestions. We feel sorry for our poor writing, however, we do invite a friend of ours who is a native English speaker from the USA to help polish our article. Due to our friend’s help, the article was edited extensively. We carefully revise the entire text of the article, improve the wording, use the current bibliography, and revise the wording in English. We hope the revised manuscript will be acceptable to you.

We concur with the view that your suggestions had a beneficial effect on the quality and readability of the article. Furthermore, we consider that they enhanced the persuasiveness and veracity of the research. We would like to express our gratitude once more for your invaluable feedback. We will continue to strive for further improvements to the article's quality.

We would be delighted to hear from you if you have further suggestions or require further information. We await your feedback with interest.

Sincerely thank you!

Yuxue Feng

[fyxgzhmu1226@outlook.com](mailto:fyxgzhmu1226@outlook.com)

**References**

1.	Bjerregaard LG, Pedersen DC, Mortensen EL, Sørensen TIA, Baker JL. Breastfeeding duration in infancy and adult risks of type 2 diabetes in a high-income country. Matern Child Nutr. 2019;15(4):e12869. Epub 2019/07/04. doi: 10.1111/mcn.12869. PubMed PMID: 31267694; PubMed Central PMCID: PMCPMC6860000.

2.	Jaddoe VW, de Jonge LL, Hofman A, Franco OH, Steegers EA, Gaillard R. First trimester fetal growth restriction and cardiovascular risk factors in school age children: population based cohort study. Bmj. 2014;348:g14. Epub 2014/01/25. doi: 10.1136/bmj.g14. PubMed PMID: 24458585; PubMed Central PMCID: PMCPMC3901421 at www.icmj.org/cio_disclosure.pdf (available on request from the corresponding author) and declare: no financial support from any organisation for the submitted work other than those listed above; no financial relationship with any companies that might have an interest in the submitted work in the previous three years; no non-financial interests or relationships that may be relevant to the submitted work.

3.	Weng L, Xu Z, Chen C. Associations of blood cadmium and lead concentrations with all-cause mortality in US adults with chronic obstructive pulmonary disease. J Trace Elem Med Biol. 2024;81:127330. Epub 2023/11/05. doi: 10.1016/j.jtemb.2023.127330. PubMed PMID: 37924610.

4.	Madrigal JM, Persky V, Pappalardo A, Argos M. Association of heavy metals with measures of pulmonary function in children and youth: Results from the National Health and Nutrition Examination Survey (NHANES). Environ Int. 2018;121(Pt 1):871-8. Epub 2018/10/22. doi: 10.1016/j.envint.2018.09.045. PubMed PMID: 30343186; PubMed Central PMCID: PMCPMC6277046.

5.	Zhang Y, Gong X, Li R, Gao W, Hu D, Yi X, et al. Exposure to cadmium and lead is associated with diabetic kidney disease in diabetic patients. Environ Health. 2024;23(1):1. Epub 2024/01/04. doi: 10.1186/s12940-023-01045-z. PubMed PMID: 38166936; PubMed Central PMCID: PMCPMC10763104.

6.	Brookheart RT, Lewis WG, Peipert JF, Lewis AL, Allsworth JE. Association between obesity and bacterial vaginosis as assessed by Nugent score. Am J Obstet Gynecol. 2019;220(5):476.e1-.e11. Epub 2019/02/02. doi: 10.1016/j.ajog.2019.01.229. PubMed PMID: 30707966; PubMed Central PMCID: PMCPMC7232937.
